# 肺癌骨转移诊断标记物研究进展

**DOI:** 10.3779/j.issn.1009-3419.2018.08.07

**Published:** 2018-08-20

**Authors:** 超 孟, 传昊 汤, 军 梁

**Affiliations:** 102206 北京，北京大学国际医院肿瘤内科 Department of Oncology, Peking University International Hospital, Beijing 102206, China

**Keywords:** 肺肿瘤, 骨转移, 标记物, Lung neoplasms, Bone metastases, Biomarkers

## Abstract

中晚期恶性肿瘤容易发生骨转移，随诊疗水平不断提升使其生存期得以延长，骨转移发生率亦随之增加。其中，肺癌是全世界癌症相关死亡的首要原因，晚期肺癌患者骨转移发生率为30%-40%。传统的肺癌骨转移诊断通常依据临床症状、X线、计算机断层扫描（computed tomography, CT）、核磁共振（magnetic resonance imaging, MRI）等影像学及病理，基于血液标记物检测作为骨转移筛查与疗效评估的探索性研究近年多有报道。本文综述了肺癌骨转移诊断标记物的研究进展。

## Abstract (en)

Bone is one of the most metastatic sites of advanced malignant tumors. With the continuous improvement of diagnosis and treatment of malignant tumors, the survival time of patients is prolonged and incidence of bone metastases also increases. Lung cancer is the leading cause of cancer-related mortality worldwide. It is estimated that the incidence of bone metastases in patients advanced lung cancer is about 30%-40%. The traditional diagnosis of bone metastases in lung cancer is based on clinical symptoms, X ray, computed tomography (CT), magnetic resonance imaging (MRI) and pathology. Recently, a large number of exploratory studies have reported blood biomarkers as indicators of bone metastasis screening and efficacy evaluation. In this review, we summarize the progress of biomarkers in diagnosis of bone metastases of lung cancer.

骨骼是中晚期恶性肿瘤常见的转移部位，在肺癌、乳腺癌及前列腺癌患者中尤其多见，可导致严重的骨骼病变，包括骨疼痛、病理性骨折、脊髓压迫、高钙血症等骨相关事件（skeletal related event, SRE），不仅严重影响患者生活质量，甚至缩短患者生存期。其中肺癌是我国发病率和死亡率居第一位的恶性肿瘤，晚期首诊肺癌骨转移发生率为30%-40%^[1]^。Santini等^[2]^对661例非小细胞肺癌患者的研究结果显示骨转移者中位生存期仅为9.5个月，且主要为溶骨性病变。因此如何早期、准确诊断肺癌骨转移，预防或避免骨相关事件发生，对肺癌患者意义重大。临床上诊断骨转移常应用影像学检查，如X线、全身骨扫描（single-photon-emission computed tomography, SPECT）、计算机断层扫描（computed tomography, CT）、核磁共振（magnetic resonance imaging, MRI）、正电子发射计算机断层显像/计算机断层扫描（positron emission tomography/computed tomography, PET/CT）等。这些检查部分存在诊断敏感性或特异性不高，导致假阳性或假阴性结果。亦或是价格昂贵，患者依从性欠佳，作为疗效评价、动态评估的工具，很难做到反复检查。骨转移血液学标志物主要包括骨形成标志物（骨源性碱性磷酸酶、Ⅰ型前胶原、骨胶素），骨吸收标志物（Ⅰ型胶原、尿吡啶啉、抗酒石酸酸性磷酸酶、Ⅰ型胶原吡啶交联终肽）及其他血清标志物（骨唾液酸蛋白、甲状旁腺素相关蛋白、微小RNA、Dickkopf-1、胰岛素样生长因子结合蛋白、血小板源性生长因子受体等）。这些标志物具有检测操作简便、可重复性好等优点，多项研究结果提示其有可能成为潜在的恶性肿瘤骨转移筛查、诊断、疗效评价的参考指标，甚至可能为独立预测和预后因子。但由于国内外尚无明确统一的检测项目、标准，多数仍停留在研究阶段，未被指南推荐应用于临床^[3, 4]^。本文对目前研究较多的肺癌骨转移血液标志物指标进行综述。

## 1 骨代谢生化标志物

骨代谢状态可从血液、尿液中检测出来，依据其发生过程的不同阶段分为骨形成标志物和骨吸收标志物两大类。

### 1.1 骨形成标志物

#### 1.1.1 骨源性碱性磷酸酶

血清总碱性磷酸酶分别由肝脏及成骨细胞合成，均为同工酶，表达程度可以反映骨的形成进程，骨源性碱性磷酸酶（bone alkaline phosphatase, BAP）由成骨细胞分泌，能够特异性反映成骨细胞的功能变化。在成骨细胞发育过程中，BAP是成骨细胞早期分化的标志，是特异的骨形成指标。本科课题组既往对130例肺癌骨转移患者的血清检测研究中发现，与对照组及无骨转移肺癌患者相比，肺癌骨转移组BAP明显升高和最佳cut-off为21.8 μg/L，其诊断敏感性、特异性为63.1%，77.0%，同时发现BAP升高不仅可提示骨转移存在，对骨转移负荷即骨转移部位的多少和骨质破坏的严重程度也有提示价值^[5]^。一项包含19项研究、3, 268例实体肿瘤患者的荟萃分析^[6]^显示骨转移组血BAP明显高于无骨转移组[(41.5±26.61) μg/L *vs* (14.49±5.52) μg/L, *P* < 0.05]。Huang等^[7]^荟萃分析了来自多中心的1, 720个样本，结果显示骨转移组BAP明显高于无转移组，此外还提示BAP升高与骨转移患者疼痛、疾病进展相关，结论与本课题组研究大致相同，但具体cut-off值并未统一。

#### 1.1.2 Ⅰ型前胶原

骨形成过程中，Ⅰ型前胶原被成骨细胞分泌到细胞外，通过细胞内外切酶作用裂解为3个片段：Ⅰ型前胶原N端前肽（N-terminal propeptide of I precollagen, PINP）、C端前肽（C-terminal propeptide of I precollagen, PICP）和Ⅰ型胶原。其中Ⅰ型前胶原是骨纤维的主要成分，被组装在类骨质中，与无机矿物质形成羟基磷灰石，不易被检测；PINP和PICP作为其代谢产物，在血液或尿液中可被检测，是Ⅰ型胶原沉积的特异性标志物，可反映骨形成水平。这两种标志物目前在乳腺癌及前列腺癌骨转移患者中研究较多，在肺癌患者中研究报道较少。PINP对乳腺癌早期骨转移诊断灵敏度，特异性均优于骨显像（64.05% *vs* 50.32%, 81.99% *vs* 61.17%）^[8]^。PINP、PICP是激素抵抗的前列腺癌骨转移患者的独立预后指标，存在这两种标记物水平升高的患者可从抑制成骨细胞药物中获益^[9]^。

#### 1.1.3 骨钙素

与BAP和Ⅰ型前胶原相比，骨钙素（osteocalcin, OC）被认为是骨基质含量最丰富和骨形成过程中产生较晚的标志物，在成骨细胞合成类骨基质时释放到细胞外基质，其中一小部分进入血液循环。破骨细胞吸收时OC会增高，反映骨形成状态，也代表骨转化水平的综合状态。有学者^[10, 11]^报道肺癌骨转移组中OC显著高于无转移组，但随后其他一些研究结果未见明显差异。Mountzios等^[12]^研究指出，因OC受到年龄、性别及肾脏功能等因素影响，可能是其不能作为肺癌骨转移预测、预后有价值指标的原因。

### 1.2 骨吸收标志物

#### 1.2.1 Ⅰ型胶原交联末端肽

骨基质的有机成分中，Ⅰ型胶原含量超过90%，骨破坏时，Ⅰ型胶原降解后释放入血，分子末端与吡啶啉形成交联物，N端与C端分别形成Ⅰ型胶原交联氨基末端肽（N-telopeptide of type Ⅰ collagen, NTX）与Ⅰ型胶原交联羧基末端肽（C-telopeptide of type Ⅰ collagen, CTX）。二者以完整的免疫原形式进入血液及尿液中，CTX其异构体受饮食影响较大。NTX浓度不受饮食影响，可较好地反映骨胶原分解及骨质溶解情况。总结多种骨转移相关分子标记物研究发现监测NTX对骨转移有预测价值，在多种实体肿瘤中，NTX与患者OS有关，高NTX与增加死亡风险相关^[7]^。关于检测血还是尿NTX，就目前研究结果，学者们存在争议，有研究^[13, 14]^显示尿NTX是提示肺癌骨转移有价值的指标。但另有学者认为尿NTX相对于血NTX受新陈代谢影响更大，血NTX能更准确的反映骨转移，Zhang等^[15]^的荟萃分析中包括14项研究，1, 279例中国肿瘤患者，结果提示血NTX与骨转移有显著的相关性。

#### 1.2.2 血清抗酒石酸酸性磷酸酶-5b

血清抗酒石酸酸性磷酸酶5b（tartrate-resistant acid phosphatase 5b, TRACP-5b）是酸性磷酸酶6种同工酶（0型-5型）其中一种，主要由破骨细胞分泌，具有降解骨基质中钙磷矿化底物的酶活性，被研究者们认为是可反映破骨细胞活性和骨吸收程度的指标^[16]^。TRACP-5b从破骨细胞释放入血，在被循环清除前已经灭活并降解成肽片段，故其血清含量不受昼夜、饮食、肝肾能影响，体内波动幅度小^[17]^，稳定性较好。Yao等^[18]^研究指出肺癌骨转移血清TRACP-5b高于无转移组[(3.50±2.23)U/L *vs* (2.09±0.72) U/L, *P* < 0.01]，这一结果与本课题组后来的研究结果相符。为避免生理性骨质疏松此指标升高的干扰，本课题组将其与另一骨吸收标志物Ⅰ型胶原吡啶交联终肽共同分析，结果显示，与单独任一指标相比，两种指标联合评估，可提高其对肺癌骨转移筛查的灵敏度和特异性（71.5%, 93.3%）。Huang等^[7]^分析了多项血清TRACP-5b与骨转移关系的研究，与恶性肿瘤无骨转移组相比，存在骨转移的高加索患者血清TRACP-5b较低，而亚洲患者血清TRACP-5b却显著升高，二者都具有统计学意义，作者考虑造成这一结果最有可能的原因是不同的种族基因和生活环境。

#### 1.2.3 Ⅰ型胶原吡啶交联终肽

Ⅰ型胶原吡啶交联终肽（type Ⅰ collagen carboxyterminal telopeptide, ICTP）是基质金属蛋白酶介导的Ⅰ型胶原代谢产物，主要存在于骨骼中，在破骨作用同时其肽段可被完整释放到血中，因其增高程度与破骨细胞活性一致，故可反映骨降解情况。既往研究结果^[19]^指出肺癌骨转移患者ICTP与CTX水平均升高，ICTP的敏感性与准确性优于CTX（75.4% *vs* 65.6%; 72.9% *vs* 68.8%），提示检测ICTP水平对肺癌患者骨转移诊断有提示作用。本课题组曾对肺癌患者检测血清BAP、TRACP-5b、ICTP，结果显示在这3种指标中，ICTP相对于BAP和TRACP-5b对诊断肺癌骨转移的敏感性，特异性最高，最佳界值为8.8 μg/L^[5]^。而Yokoyama等^[20]^研究结论指出在肺癌患者中ICTP作为诊断骨转移时界值应为6.4 ng/mL，不同于乳腺癌患者骨转移诊断时的4.5 ng/mL。

## 2 其他可反映肺癌骨转移的标志物

### 2.1 骨唾液酸蛋白

骨唾液酸蛋白（bone sialoprotein, BSP）是骨骼细胞外基质中的一种酸性糖蛋白，由成骨细胞、破骨细胞和软骨细胞合成，通过与整合素识别介导新血管的生成和肿瘤细胞的粘附、迁移，还与肿瘤细胞逃避免疫监视相关。Zhou等^[21]^对105例Ⅲ期非小细胞肺癌患者建立了一个预测骨转移发生的分子模型，包含BSP在内的4种标志物，结果显示，此分子模型对诊断骨转移的敏感性和特异性分别为85.7%和66.7%。其中在骨转移患者肿瘤细胞中BSP的表达水平高于原发肿瘤，故BSP表达水平高的原发肿瘤更倾向发生骨转移。

### 2.2 甲状旁腺素相关蛋白

甲状旁腺素相关蛋白（parathyroid hormone related protein, PTHrP），存在多种不同的片段，在多种组织中存在，具有广泛的生理作用。多种类型肿瘤组织中发现了PTHrP表达，不同于正常组织，肿瘤组织是持续大量的分泌PTHrP，使其直接或间接作用于破骨细胞，因其破骨细胞分化、活跃，进而导致骨质吸收。高钙血症的肺癌患者PTHrP水平升高，其浓度 > 150 pmol/L与骨转移发病率有显著相关性（71.4%），且中位生存期较短（1.4个月）^[22]^。Kuo等^[23]^对肺癌患者细胞系的研究发现，PTHrP表达与miR-33a水平呈负相关，此微小RNA可抑制PTHrP基因转录，具有反映骨重吸收潜在标记物的价值，还可作为治疗的靶点。

### 2.3 肺癌骨转移相关微小RNA、基因

微环境改变在肿瘤的发生、发展过程中起到重要作用，骨转移的发生也不例外。骨转移作为骨骼特殊发育过程，需要骨髓中的循环肿瘤细胞参与，它们可以改变骨骼微环境中的破骨细胞和成骨细胞功能，改变骨正常形成。这些过程均需要肿瘤细胞与骨微环境相关作用，从而影响基因的表达。研究^[24, 25]^显示，微小RNA（small non-coding microRNAs, miRNAs）作为基因表达的主调控者，参与骨转移形成的多个方面，包括肿瘤细胞从原发部位逃逸、转移到骨组织、转移瘤与肿瘤基质细胞相互作用等环节。在肺癌骨转移基础研究中发现，miR-33a、miR-326表达与PTHrP、PINP水平相关^[24]^，癌症基因组图谱分析则显示烯醇化酶1、钙磷蛋白等基因的水平对肺癌骨转移也有监测作用^[26]^，且比蛋白表达要更加提前，这些指标的特异性、敏感性仍需要进一步研究、验证。

### 2.4 Dickkopf-1与胰岛素样生长因子结合蛋白3

Dickkopf-1（DDK1）是一种分泌蛋白，通过与低密度脂蛋白受体相关蛋白5/6结合抑制Wnt信号通路^[27]^，导致骨吸收，抑制骨形成，促进恶性肿瘤骨转移^[28]^。Pang等^[29]^下调DDK1表达后，肺癌骨转移细胞系在体外的繁殖、细胞迁移及侵袭能力均受到抑制，凋亡率增加；在体内骨转移的发生率也下降。胰岛素样生长因子结合蛋白3（insulin like growth factor binding protein 3, IGFBP-3）是胰岛素样生长因子结合蛋白家族的一种，可调节胰岛素样生长因子（insulin-like growth factor, IGF）水平，从而影响肿瘤的发生、发展。Sun等^[30]^分析了有无骨转移肺癌患者13种细胞因子后发现，骨转移组IGFBP-3水平明显高于无转移组（*P*=0.014），进一步研究显示，IGFBP-3水平与促进血管再生的血管内皮生长因子（vascular endothelial growth factor, VEGF）水平呈正相关性，二者协同作用于骨转移。虽然研究^[31]^提示这两种标记物对肺癌骨转移有诊断价值，但其特异性仍不充分。

## 3 结语

综上所述，血清骨转移相关标志物具有取材简单，重复性好等优势。但是我们同时也发现，在不同种族和不同瘤种之间，包括不同的检测平台，存在cut-off值不统一的问题。此外单个标志物的特异性、敏感性有待提高，联合检测和分析的结果尚需临床进一步验证。研究者仍在继续探寻新的骨转移相关标志物及检测方法，以期实现精准医疗时代的肺癌骨转移早期筛查，确诊骨转移的动态监测，指导临床精准用药与全程管理，预防骨相关事件发生，从而达到提高患者生存质量，最终延长生存的目的。
